# In Vitro and In Silico Study on the Impact of Chlorogenic Acid in Colorectal Cancer Cells: Proliferation, Apoptosis, and Interaction with β-Catenin and LRP6

**DOI:** 10.3390/ph16020276

**Published:** 2023-02-13

**Authors:** Laura Catalina Vélez-Vargas, Gloria A. Santa-González, Diego Uribe, Isabel C. Henao-Castañeda, Johanna Pedroza-Díaz

**Affiliations:** 1 Grupo de Investigación e Innovación Biomédica, Facultad de Ciencias Exactas y Aplicadas, Instituto Tecnológico Metropolitano, Medellin 050012, Colombia; 2 Productos Naturales Marinos, Facultad de Ciencias Farmacéuticas y Alimentarias, Universidad de Antioquia, Medellin 050010, Colombia

**Keywords:** chlorogenic acid, colorectal cancer, apoptosis, proliferation, β-catenin, LRP6

## Abstract

Colorectal cancer mortality rate and highly altered proteins from the Wnt/β-catenin pathway increase the scientific community’s interest in finding alternatives for prevention and treatment. This study aims to determine the biological effect of chlorogenic acid (CGA) on two colorectal cancer cell lines, HT-29 and SW480, and its interactions with β-catenin and LRP6 to elucidate a possible modulatory mechanism on the Wnt/β-catenin pathway. These effects were determined by propidium iodide and DiOC6 for mitochondrial membrane permeability, MitoTracker Red for mitochondrial ROS production, DNA content for cell distribution on cell cycle phases, and molecular docking for protein–ligand interactions and binding affinity. Here, it was found that CGA at 2000 µM significantly affects cell viability and causes DNA fragmentation in SW480 cells rather than in HT-29 cells, but in both cell lines, it induces ROS production. Additionally, CGA has similar affinity and interactions for LRP6 as niclosamide but has a higher affinity for both β-catenin sites than C2 and iCRT14. These results suggest a possible modulatory role of CGA over the Wnt/β-catenin pathway in colorectal cancer.

## 1. Introduction

Colorectal cancer (CRC) is the third leading cause of cancer-related death worldwide and the fourth with more incidence [1]. Like other types of cancer, the primary treatment for CRC is surgery; nevertheless, most patients are asymptomatic until the tumor progresses and must be treated with a combination of chemotherapy, radiotherapy, and biotherapy [2]. Despite advances in prevention, screening, and chemotherapy, the 5-year survival rate in CRC is about 11% of all cancer [3]. For this reason, developing new treatment regimens for managing these patients is mandatory [4]. 

Chlorogenic acid (CGA) is a polyphenol widely distributed in nature. It is an ester of caffeic acid with quinic acid, whose antiproliferative effect in CRC has been demonstrated [5]. Previous studies using in vitro colon cancer models have shown that CGA decreases viability and modulates the proliferative and migratory capabilities of Caco-2 [6], HT-29, SW480, and SW620 [7,8] cells at EC50 758 uM and IC_50_ 8114 μg/mL, 686.6 μg/mL, and 828.6 μg/mL, respectively. Likewise, it has been shown that CGA diminishes the viability of cancer cells derived from liver [9,10,11], breast [12,13], lung [14,15], blood [16,17], bone [18], and kidney [19] tissues, suggesting the potential of CGA for the modulation of biological mechanisms involved in cancer cell survival.

Wnt/β-catenin is a highly conservative signaling pathway that plays a crucial role in embryonic development and regulates the self-renewal and homeostasis of various adult tissues [2]. More than 94% of CRC cases have at least an altered protein from the Wnt/β-catenin signaling pathway by a genetic and epigenetic mechanism [20]. However, early APC mutations are acquired in over 80% of colon cancer patients, leading to the cytosolic accumulation of β-catenin, an intracellular signal transducer in transcriptional regulation that, in combination with TCF/Lef1, promotes proliferation and inhibits apoptosis [21]. Moreover, recently, we reported the modulatory effect of CGA treatment ong SW480 and HT-29 cell, by decreasing the transcriptional activation of the Wnt pathway at similar proportion as the selective pathway inhibitor iCRT14 [8].

**Figure 1:**
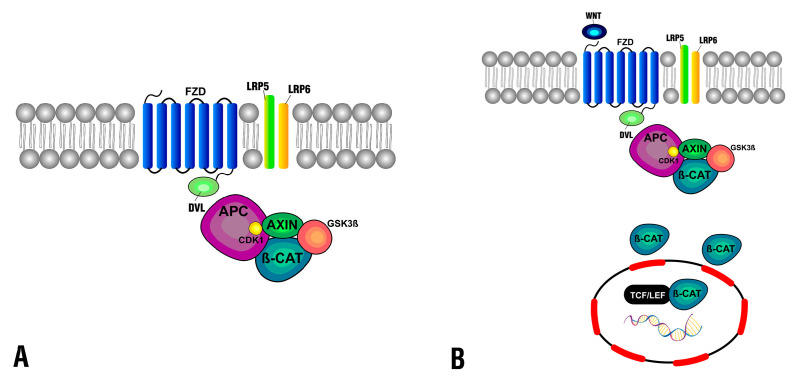
Canonical Wnt/β-catenin signaling pathway. (**A**) Regulated state when the destruction complex controls β-catenin levels at the cytoplasm. (**B**) Dysregulated state where the presence of Wnt ligands and the interaction with membrane receptors displace the destruction complex and β-catenin accumulates at the cytoplasm, and then translocated to the nucleus.

In normal physiological conditions, β-catenin degrades in the cytoplasm by the destruction complex consisting of axis inhibition protein (AXIN), adenomatosis polyposis coli (APC), casein kinase 1 (CDK1), and glycogen synthase kinase 3β (GSK3β) (Figure 1A). As a consequence of Wnt ligands binding to the Frizzled protein receptor at the membrane and its coreceptor LRP5/6, the destruction complex function becomes displaced, which leads to the accumulation of β-catenin and its translocation to the nucleus (Figure 1B), consequently triggering the CRC [22]. 

LRP6 possesses extracellular and intracellular domains. The extracellular domain is necessary for the interaction with Wnt ligands and also with Dickkopf-related protein 1 (DKK1) [23], a protein that antagonizes the P3E3P4E4 domain by competitive binding, disrupting the initiatory complex Frizzled–LRP6 [24]. For this reason, inhibiting LRP6 can be a potential solution for diseases such as osteoporosis, Alzheimer, cancer, and neurodegeneration [23]. It has been found that niclosamide is a potent Wnt/β-catenin inhibitor by inducing LRP6 degradation in HEK293 cells [25]. Furthermore, it has been observed that niclosamide suppresses the growth of HCT116, LoVo, SW620, and HT-29 cell lines [26], and additionally induces cell apoptosis [27]. 

**Figure 2:**
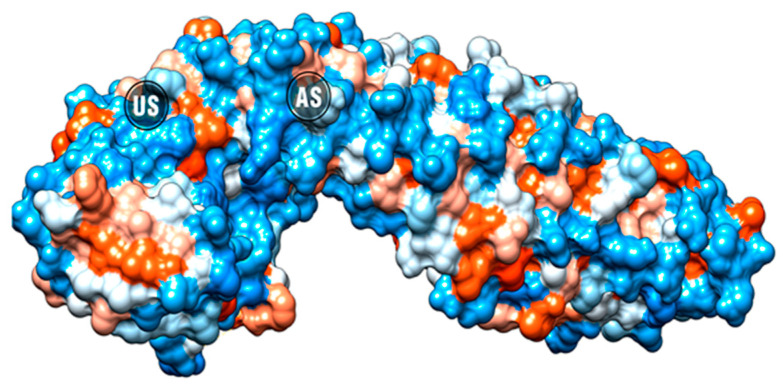
β-catenin surface with the position of the allosteric site (AS) and the TCF4 binding site (US) highlighted.

The WNT pathway inhibition could also be assessed by blocking the most downstream component, the interaction between β-catenin and T-cell factor 4 (TCF4) at the nucleus. This inhibition can be effectively achieved regardless of the mutations in the upstream components [28]. iCRT3, iCRT5, and iCRT14 effectively inhibit β-catenin–TCF4 interaction while allowing β-catenin binding with E-cadherin at the cell–cell adherens junctions, which is essential for the prevention of metastasis [29]. β-catenin has two binding sites (Figure 2), the well-known union site (US) between β-catenin and TCF4 [30] and the recently reported allosteric site (AS) [31].

The CRC model selected for this research consists of two cell lines with different truncating mutations in the APC gene. In SW480, the mutation at the 1338 residue changes the APC domain that interacts with β-catenin for ubiquitination and degradation, while in HT-29, the truncating mutation in the 1555 residue maintains the regulatory effect of the APC protein over β-catenin [32,33,34].

Proliferation and apoptosis are processes highly involved in cancer development and important to cancer therapy [35,36]. Despite the multiple studies evaluating the proliferation and apoptosis in colorectal cell lines, to the best of our knowledge, there are no available reports comparing the effects of CGA on SW480 and HT-29 cell lines, in which differences in the Wnt/β-catenin pathway have been reported. Although there are studies describing that CGA regulates Wnt/β-catenin signaling [37,38], none of them discriminate which targets are involved in the mechanism.

This study aims to determine the effect of CGA in proliferation and apoptosis on two colorectal cancer cell lines and its interactions with β-catenin and LRP6 to elucidate a possible modulatory mechanism of CGA on CRC in the context of Wnt/β-catenin pathway. 

## 2. Results

### 2.1. CGA Treatment Affects Cell Viability 

**Figure 3:**
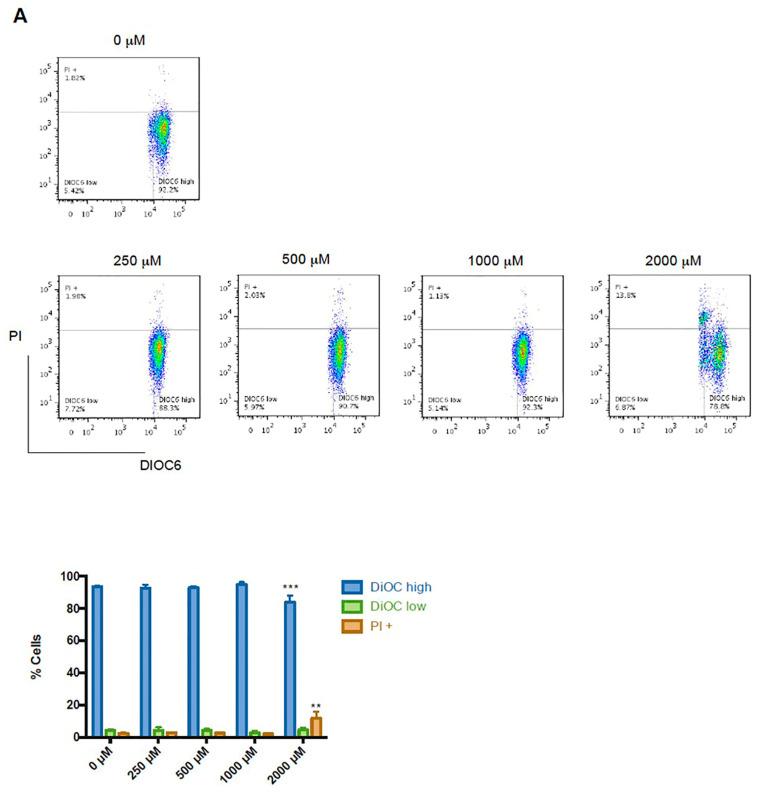

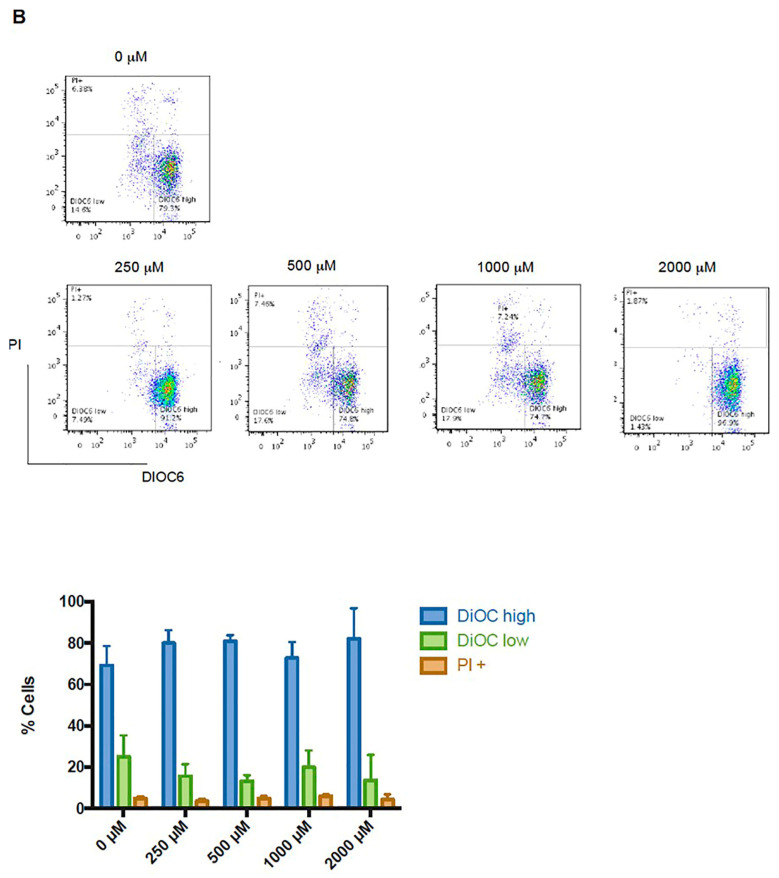
Cytotoxic effect of CGA in colon cancer cells. (**A**) SW480 and (**B**) HT-29 cells were treated with CGA at indicated concentrations. Mitochondrial membrane potential was measured with DiOC6 and cell membrane by PI intake and analyzed using flow cytometry. DiOC6 high: live cells with high membrane polarization; DiOC6 low and PI negative: cells with membrane depolarization; PI+: dead cells. The figure shows a representative histogram of flow cytometry analysis and bar graphs for quantification for each cell line. Two-way ANOVA for DiOC high, DiOC low, and PI+ populations shows the difference concerning untreated cells, where ** *p* ≤ 0.01, *** *p* ≤ 0.001.

Mitochondrial polarization changes and loss of cell membrane integrity are common indicators of cell death. SW480 and HT-29 colon cancer cells were treated with different concentrations of CGA to determine the cytotoxic effect. Flow cytometry was used to quantify the DiOC6 retention and PI uptake. In SW480 cells, 2000 µM of CGA induced a significant decrease in DiOC6 high population with a related increase in PI uptake (Figure 3A). These results indicate a reduction in cell viability because mitochondrial dysfunction is directly involved in the intrinsic apoptotic pathway. Under the same conditions, HT-29 cells do not exhibit a significant difference (Figure 3B).

### 2.2. CGA Induces Mitochondrial Reactive Oxygen Species (ROS) Production

**Figure 4:**
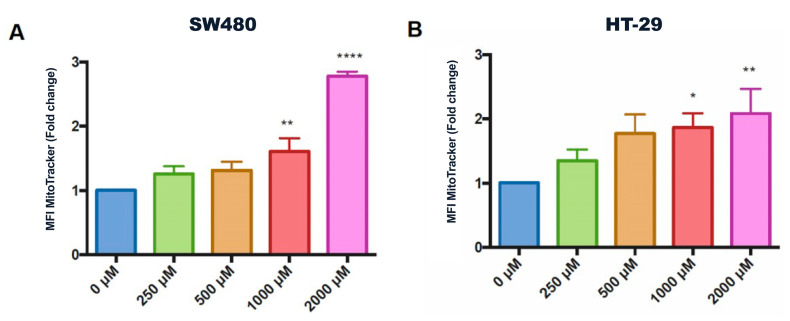
Effects of CGA on mitochondrial ROS production in colorectal cancer cells. (**A**) SW480 and (**B**) HT-29 cells were treated with CGA as indicated concentrations. MitoTracker Red CMXRos, a red fluorescent dye that stains mitochondria in live cells and fluoresces upon oxidation, was used to examine mitochondrial changes in ROS levels. One-way ANOVA for concentration effect, differences with respect to untreated cells, where * *p* ≤ 0.05, ** *p* ≤ 0.01, **** *p* ≤ 0.0001.

ROS production was assessed to investigate the mode of action of CGA on colon cancer cells. Figure 4 shows the quantification of the mean fluorescence intensity of MitoTracker Red CMXRos in SW480 and HT-29 cells, with a dose-dependent increase in ROS detection. SW480 cells showed higher ROS production after CGA treatment. Mitochondrial ROS increase can depolarize the mitochondrial membrane, as was observed in the previous results for SW480, which could result in increased activation of proapoptotic molecules, such as Caspase-3 [39].

### 2.3. CGA Produces DNA Fragmentation Preferentially in SW480 Cells

**Figure 5:**
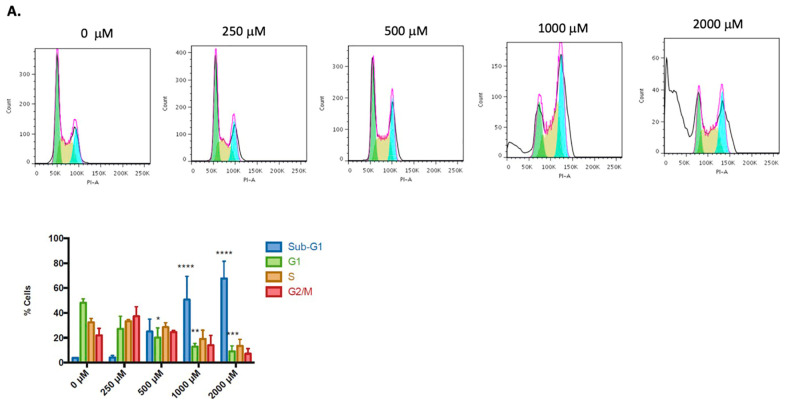

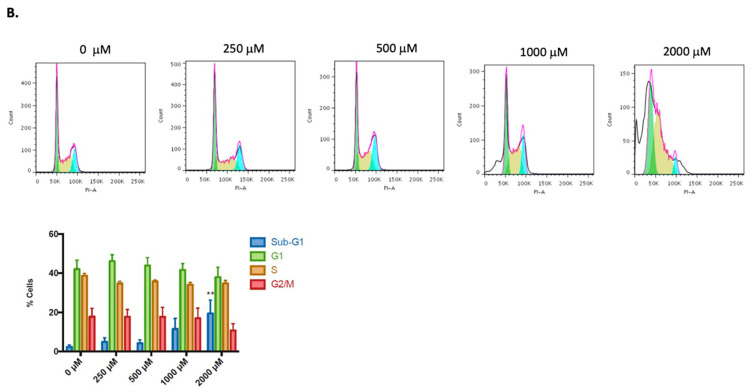
Cell cycle distribution after CGA treatment (24 h) in colorectal cancer cell lines SW480 (**A**) and HT-29 (**B**). The figure shows a representative histogram and bar graphs of flow cytometry analysis of the cell cycle distribution in the different phases. Two-way ANOVA for sub-G1, G1, S, and G2/M populations, displaying the difference with respect to untreated cells, where * *p* ≤ 0.05, ** *p* ≤ 0.01, *** *p* ≤ 0.001, **** *p* ≤ 0.0001.

An analysis of the cell cycle distribution was used to describe the antiproliferative effect of CGA in colorectal cancer cells (Figure 5). Each cell line had a typical cell cycle distribution, with most cells in the G1 phase. At 500 μM and higher doses, CGA-treated cells showed a significant drop in the G1 phase percentage compared with untreated cells. This decrease in G1 cells occurred with an increased percentage of cells in the sub-G1 phase. The characteristic sub-G1 peak could be fragmented DNA marked with low-level DNA fluorescence, suggesting apoptotic cell death, as has been reported by other authors [40,41,42]. DNA fragmentation is more significant in SW480 (*p* < 0.001, Figure 5A) than in HT-29 cells (*p* < 0.01, Figure 5B).

### 2.4. Molecular Docking

**Figure 6:**
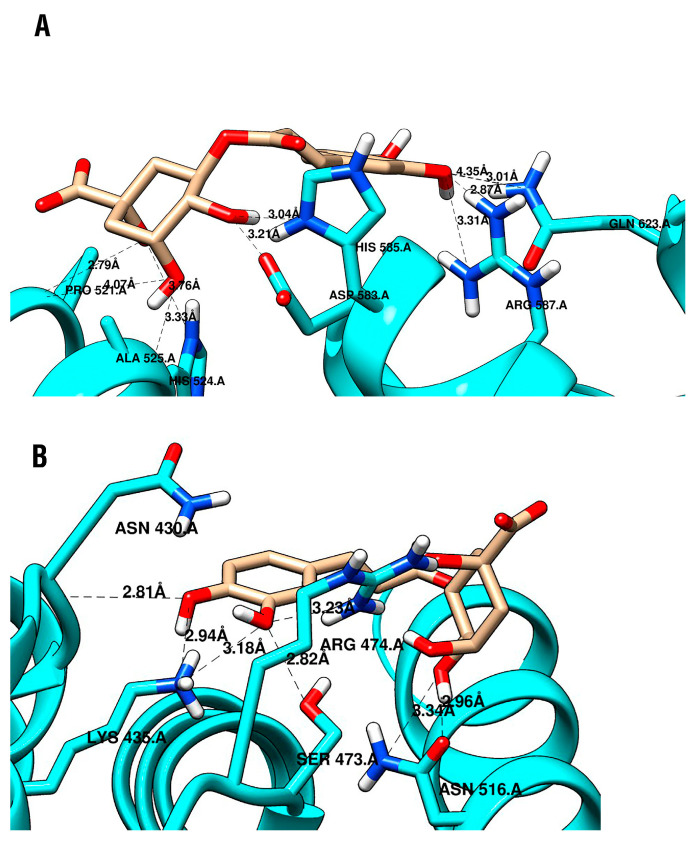
CGA interacting with β-catenin (**A**) at the allosteric site and (**B**) at the binding site.

**Figure 7:**
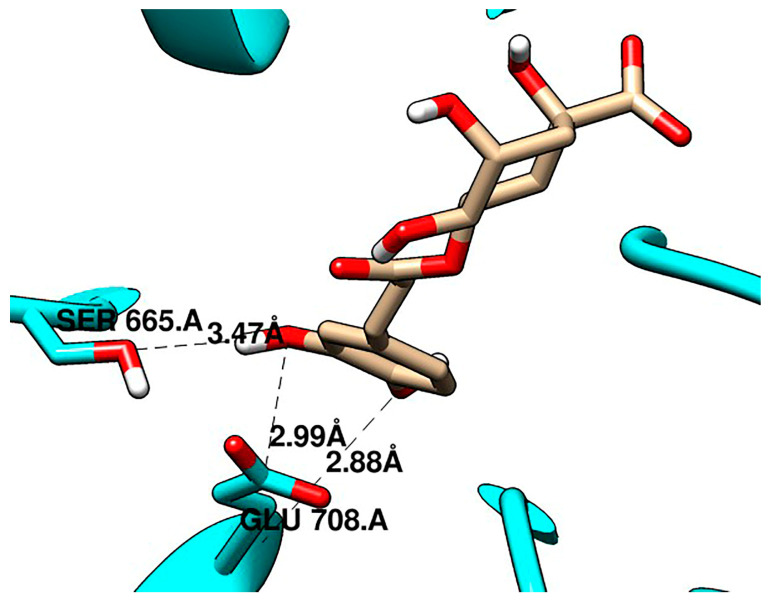
CGA interacting with LRP6 at the E3 site.

Molecular docking studies were performed between CGA and two crucial proteins in the WNT/β-catenin pathway, and known inhibitors were used for validation. CGA showed a higher affinity for both sites of β-catenin than C2 and iCRT14 (Table 1) and a similar affinity for LRP6 E3 as niclosamide (Table 2). In the allosteric site of β-catenin, C2 showed an affinity of −4.6 kcal/mol, while CGA was −5.5 kcal/mol. Particularly, CGA presented more hydrogen bonds with the β-catenin sites than the validation compounds, as shown in Figure 6 and Figure 7. 

## 3. Discussion

Although different studies have reported several biological properties of CGA [43,44], there still needs to be clear evidence about the role of this natural compound in CRC. In the present study, we showed that CGA induced ROS production and mitochondrial hyperpolarization, which results in the DNA fragmentation and reduced cell viability of colon cancer cells, which harbors alterations in the Wnt/β-catenin pathway. Additionally, we explore the interaction of CGA with β-catenin and LRP6 by in silico approaches to rationalize if the in vitro biological results could be potentially related to the possible modulation of the Wnt/β-catenin pathway. 

According to our results, the concentrations of CGA required to achieve the desired biological effect in colorectal cancer cells are higher than the concentrations absorbed from food, and also in other reports that use CGA in lower doses [45,46]. However, generally, they do not determine the IC_50_ to select the working doses [11,14,17]. The advantage of using the concentrations suggested by the IC_50_ curves is that the sensitivity of every model is considered.

Limitations of CGA, such as low absorption and variable bioavailability, could affect scale-up to the in vivo study phase. Strategies to improve its pharmacokinetic profile could be a perspective of this work. For instance, some studies have shown that structural modifications of polyphenols, such as resveratrol and curcumin, increased their cytotoxic, antiproliferative, and proapoptotic effect [47,48]. Particularly, we have initiated a new project where we synthesize derivatives of CGA that showed a lower IC50 than CGA. However, it is still unknown if it could be due to a better pharmacokinetic profile or a higher derivative activity [49].

In this work, we observed an incremented ROS production in both the SW480 and the HT-29 cell lines (Figure 4) in the treatment with CGA, which results in mitochondrial hyperpolarization and an increase in sub-G1 cell population (Figure 5), suggesting DNA fragmentation (a hallmark of apoptotic cell death). Even though physiological levels of ROS play important roles in promoting normal cellular processes [50], ROS overproduction has been related to the oxidation of molecules, such as nucleic acids, lipids, carbohydrates, and proteins [51], which results in the alteration of the vital process, including cell-death-related mechanisms [52,53]. 

The results showed that CGA reduces the cell viability of the SW480 cells while not exhibiting a significant effect on the HT-29 cells (Figure 3); this behavior could be related to the enhancement in ROS production that could cause an alteration in the antioxidant balance of the cells. Although ROS production is observed at 1000 μM in both cell lines, the cell viability was not affected, probably because no damage was still involved in the macromolecules. Meanwhile, at 2000 μM, there is already a loss of cell homeostasis and induction of cell death [54].

Interestingly, different phytochemicals have been shown to possess pro-oxidant properties in the context of cancer through the induction of ROS accumulation in cancer cells and the subsequent activation of apoptotic cell death [51]. This is the case of complex mixtures of phytochemicals, such as *Mangifera indica L.* peel extracts [55], *Passiflora edulis f. flavicarpa* leaf extracts [56], *Vaccinium meridionale* Swartz juice [57], fermented nondigestible fraction from spent *Coffea arabica* grounds [58], fermented nondigestible fraction from *Moringa oleifera* leaves [59], *Persea americana* pulp extracts [60], and independent natural compounds, including quercetin [61], procyanidins [62], cannabidiol [63], resveratrol [64], curcumin [65], catechins [66], p-coumaric acid [67], kahweol [68], and CGA [6,69,70], among others. 

To retain their malignant phenotypes, cancer cells contain higher levels of ROS and a different redox state from their normal counterparts. Because of this, cancer cells are more susceptible than normal cells to an increase in ROS generation brought on by different agents, including polyphenols [71]. Polyphenols have been shown to affect the redox status in varying ways, depending on the dose of polyphenols and the physiologic context of the interaction [72]. In cancer cells, a different mechanism of ROS induction by polyphenols has been explored, including pro-oxidant properties in systems containing redox-active metals [73,74,75], inhibition of endogenous antioxidants, and alteration in the electron transport chain [76,77]. In all cases, the mechanism through which polyphenols induce ROS production depends on concentration, structure, cell type, and experimental design.

The differences observed in the cell lines in response to CGA treatment, being SW480 more susceptible than HT-29 cells (Figure 3, Figure 4 and Figure 5), could be explained by its genetic and epigenetic backgrounds, which have a direct impact on the constitutive activation of signaling pathways involved in colon cancer onset and progression, such as TP53, KRAS, BRAF, Wnt/β-catenin, among others [78,79]. Both HT-29 and SW480 have microsatellite stable (MSS) phenotypes but differ in CpG island methylator phenotype (CIMP) and consensus molecular subtypes (CMS), being HT-29 CIMP+ and CMS3 (metabolic) with a moderately active Wnt/β-catenin pathway and, SW480 CIMP- and CMS4 (mesenchymal) with an upregulation of molecules involved in epithelial to mesenchymal transition and matrix remodeling [79,80]. Interestingly, both cell lines harbor truncating mutations in the APC gene that are tightly related to their CMS status but have a different impact on Wnt/β-catenin pathway activation. On the one hand, SW480 cells have a truncating mutation at residue 1338 in one allele of the APC gene, where the catenin inhibitory domain is located for β-catenin ubiquitination and degradation. It has been suggested as a requirement to drive high basal levels of Wnt/β-catenin pathway activity; on the other hand, HT-29 cells have a truncating mutation at residue 1555 of the APC gene, but retain the catenin inhibitory domain and the β-catenin binding sites (15RA–15RD and 20R1–20R3), which allows maintaining the regulatory effect of the APC protein over β-catenin in this cell line [32,33,34]. This is important, considering that APC mutations have been shown to cooperate with BRAF [81] and KRAS mutations [82,83] for the development of colon cancer, which are also genetic alterations present in HT-29 and SW480 cells, respectively [79]. Additionally, differences in the levels and activity of proteins of the detoxification machinery of the cells have been reported, including the ATP-binding cassette subfamily members ABCB1 and ABCG2, which are involved in the efflux of xenobiotics from cells, such as polyphenols [84], as well as the expression and activity of enzymes involved in the glucuronidation of free hydroxyl groups present in the chemical structures of polyphenolic compounds [85,86,87], increasing their polarity and water solubility for their excretion [88]. All these dissimilarities between SW480 and HT-29 could explain that despite ROS production being equivalent between both cell lines, the biological effect, including cell cycle distribution, presents significant differences.

Finally, to explore whether the in vitro biological results could be potentially related to Wnt/β-catenin pathway modulation, considering that the negative regulation of the pathway by natural products has been associated with the activation of cell-death-related mechanisms [89,90,91,92,93,94,95,96,97,98,99,100,101,102,103,104,105,106,107,108], molecular docking approaches were performed to analyze the interaction of CGA with crucial proteins in the Wnt/β-catenin pathway, named β-catenin and LRP6. In this manner, it was observed that CGA could interact with an allosteric site on the surface of β-catenin and with the binding surfaces of β-catenin involved in the interaction with the transcription factor TCF (Figure 6), and also with ectodomain 3 of LRP6 (Figure 7). It is important to note that all these interactions showed similar affinities, and in some cases even higher affinities, when compared with the validation compounds we used for the analysis (Table 1 and Table 2). 

At the well-known β-catenin binding site, it has been reported that the residues Lys435 and Arg469 form a salt bridge. Additionally, it has been predicted that the compound UU-T01 binding to β-catenin around the residues Lys436, Arg469, and Lys508 and UU-T02 forms cation-π interactions with Arg474 and Arg515 [30]. Besides, docking studies predicted GB1874 binding to the β-catenin residues Arg469, Lys508, Asn426, Arg515, Arg474, and Arg435 [28]. Our study confirmed the salt bridge between Arg469 and CGA and the π interactions between Arg474 and Arg515 with iCRT14.

The binding pocket of DKK1 involves the Glu663, Ser665, Tyr706, Asp748, Lys770, Leu810, His834, Trp850, and Tyr875 residues of LRP6 as hot spot regions of this protein. Specifically, it formed hydrophobic bonds with Leu810, Asp811, Pro833, Tyr706, and Arg639, while it formed hydrogen bonds with Glu663, Glu708, Arg792, Asp811, Thr812, Asn813, Asp830, Leu832, and Arg1184 [24]. Our study observed the hydrophobic interactions between Leu810 from LRP6 with niclosamide and CGA, the hydrogen bonds between Glu708 and CGA, and Asp811 with niclosamide.

Previous studies have shown the potential of small molecule inhibitors, including C2 [31] and iCRT14 [29], and other molecules, to negatively regulate the Wnt/β-catenin pathway by the interaction with β-catenin and LRP6 proteins. Recently, the discovery of a small-molecule inhibitor (C2) has been reported. C2 targets an allosteric site on the surface of β-catenin in armadillo domain 8–10 and induces the proteasomal degradation of the protein. This results in the downregulation of Axin1, CyclinD1, and TCF4 proteins expression and the subsequent reduction in cell viability and tumor growth, even in the context of APC mutations [31]. Likewise, iCRT14 inhibits the direct interaction of β-catenin with TCF at the nucleus, interfering with its activity as a transcriptional activator, causing a downregulation of AXIN2, CCND1, and C-MYC gene expression and the inhibition of cell proliferation and invasion [29]. On the other hand, niclosamide suppressed the LRP6 expression in TNBC MDA-MB-231 cells and ER-positive breast cancer T-47D cells and inhibited breast cancer cell proliferation with IC_50_ values less than 1 mM [25]. Altogether, these observations support our hypothesis regarding the possible modulation of the Wnt/β-catenin pathway by CGA, even in the context of APC truncating mutations, considering the interaction of this molecule (Table 2) with β-catenin (Figure 6) and LRP6 (Figure 7), similarly as C2, iCRT14, and niclosamide. These findings are of great significance in the context of CRC, considering that APC mutations are an early and critical driver in the stepwise progression from adenoma to carcinoma [109], and also because the intestinal stem cell niche provides large amounts of Wnt ligands and amplifiers, which cooperates with intrinsic alterations of Wnt pathway members for the onset and progression of CRC [110]. 

As mentioned before, the Wnt/β-catenin pathway is implicated in the development of CRC [21]; hence improving targeted therapies against this pathway is mandatory for its translation to clinical practice [111,112,113]. In this regard, natural products showed the potential to modulate several signaling pathways involved in cancer development, including Wnt/β-catenin, through the downregulation of β-catenin and Wnt-target genes expression; the modulation of β-catenin phosphorylation, ubiquitination, and proteasomal degradation; and the inhibition of its nuclear translocation, among other mechanism [114,115], which results in the activation of cell-death-related mechanisms [89,90,91,92,93,94,95,96,97,99,100,104,108,115], supporting our hypothesis regarding the possible modulation of the Wnt/β-catenin pathway by CGA. 

## 4. Materials and Methods

### 4.1. Materials

SW480 and HT-29 cells were commercially obtained from ATCC (Manassas, VA, USA). Cell culture reagents, including Dulbecco’s Modified Eagle Medium (DMEM), fetal bovine serum (FBS), antibiotics, and trypsin-EDTA, were purchased from Gibco (Grand Island, NY, USA). Chlorogenic acid was purchased from Sigma-Aldrich (St. Louis, MO, USA).

### 4.2. Cell Culture

The colon cancer cell lines SW480 and HT-29 were grown in DMEM supplemented with 5% FBS and RPMI media supplemented with 10% FBS, respectively. To complete the medium, 100 g/mL penicillin and 100 g/mL streptomycin were added. The cell cultures were kept at 37 °C in a humidified incubator with 5% CO_2_ and 95% air. Cells were evaluated regularly under a microscope for proper morphology and adhesion and were subcultured before the confluence.

### 4.3. Treatment Outline

Colon cancer cell lines were plated in 6-well plates at a concentration of 2.5 × 105 cells/mL. Cells were cultured under standard conditions, and after 24 h, to ensure adhesion and exponential growth, cells were treated for 24 h with doses of 250, 500, 1000, and 2000 µM of CGA [7]. Cells were trypsinized, pelleted, and analyzed by flow cytometry for different tests to evaluate the biological effect after treatments. Data reported included at least three separate experiments per treatment group.

### 4.4. Cell Viability

As a measure of cell viability, the cytoplasmic membrane’s integrity and mitochondrial membrane permeability changes were examined using propidium iodide (Sigma, P4170) and DiOC6 (Molecular Probes D273), respectively. Cells were pelleted and stained with 1 mg/mL PI and 50 nM DiOC6 and incubated for 30 min at room temperature to assess the incorporation of the dyes after CGA treatments. Flow cytometry was used to analyze 10,000 events with BD LSRFortessa. FlowJo was used to calculate the mean fluorescence intensity (MFI).

### 4.5. Mitochondrial ROS Production

Mitochondrial ROS production was evaluated to delimit the mechanism of cell death induction. Treated colon cancer cells were dyed with 3 μM MitoTracker Red (Invitrogen, M7512) and stored at room temperature for 20 min. After that, cells were washed twice in phosphate-buffered saline and analyzed by flow cytometry (BD LSRFortessa). The mean fluorescence intensity (MFI) of MitoTracker was calculated using FlowJo.

### 4.6. DNA Content

DNA content was analyzed to determine the cell distribution in cell cycle phases. CGA-treated cells were collected and centrifuged. Afterward, the cell pellet was fixed in 70% cold ethanol for 1 h. Permeabilized cells were incubated with 100 μg/mL of RNase (Sigma, R5000), labeled with 100 μg/mL of propidium iodide (Sigma, P4170), for 30 min and analyzed by flow cytometry (BD LSRFortessa). FlowJo was used to analyze the distribution of cell cycle phases.

### 4.7. Molecular Docking

#### 4.7.1. Ligand Selection and Preparation 

Chlorogenic acid anion and validation ligands (C2, iCRT14, and niclosamide) were built and prepared using GaussView 5 [116] and Gaussian 09 [117] with B3LYP-6-31++G** approximation. All ligands were prepared using AutoDock Tools (ADT) [118]. 

#### 4.7.2. Protein Preparation

The structures of the studied proteins β-catenin (PDB code 2GL7) and LRP6 (PDB code 3S2K) were prepared using UCSF Chimera 8 [119] and ADT, and they were used without water molecules. Polar hydrogen atoms were automatically added to the protein, also AD4 type of atoms and Gasteiger charges. 

Molecular docking was carried out using AutoDock Vina [120] with the parameters shown in Table 3. The pose with the best affinity for each site was chosen, and a visual inspection of the interactions was performed using a Discovery Studio Visualizer (BIOVIA), PLIP [121], and UCSF Chimera.

The parameters described in Table 3 were established according to the literature. β-catenin has two binding sites; the well-known union site (β-catenin US) is located in the most crucial interaction between the residue Asp16 from TCF4 and Lys435 and His470 of β-catenin [30]. For this reason, the NZ atom from Lys435 was chosen as the center of the grid for β-catenin US. On the other hand, an allosteric site of β-catenin (β-catenin AS) was recently reported, which included the residues Pro521, Arg528, and Asp583 [31], and the atom ND1 from His524, located near the center between the three reported residues, was chosen. On LRP6, the grid’s center was chosen from the coordinates of Gly227 from DKK1.

### 4.8. Statistical Analysis

ANOVA, followed by Fisher’s protected least significant difference (LSD) tests, was carried out to calculate statistical differences among nontreated cells and different doses of the treatments with CGA. *p* ≤ 0.05 was considered statistically significant. Data represent the results of a minimum of three independent experiments. Results are expressed as mean ± standard error of the mean (SEM). For graphs and analysis, the GraphPad Prism software was employed.

## 5. Conclusions

Despite the significant advances in the diagnosis of CRC, current treatments confer limited benefit, which makes this disease the third leading cause of cancer death. CRC development and progression involve altering regulatory mechanisms in one or more members of the Wnt/β-catenin signaling pathway. For this reason, identifying substances capable of modulating the Wnt/β-catenin signaling has been a significant effort for the scientific community. It is worth noting that some new compounds have recently been described as inhibitors of different components of this signaling pathway. Particular attention has been given to polyphenols. However, a long way still must be paved to achieve treatment success for CRC. CGA could be a potential coadjuvant in CRC therapy. In the present study, the induction of mitochondrial hyperpolarization, DNA fragmentation by CGA, and interactions between CGA with β-catenin and LRP6 suggest possible modulation of the Wnt/β-catenin pathway. Differences in sensitivity between SW480 and HT-29 would be related to the basal transcriptional activity of this signaling pathway in two lines. Our results provide an exciting starting point on the effect of CGA in the context of CRC and the possible modulation of the Wnt pathway. 

Additional research is needed for a more in-depth understanding of this mechanism. For instance, future research regarding Wnt/β-catenin signaling in CRC should focus on (1) achieving a deeper understanding of crosstalk among the AKT/PI3K, NOTCH, mTOR, and Wnt/β-catenin pathways; (2) optimizing and evaluating other natural compounds as Wnt/β-catenin inhibitors while also being highly selective to avoid unnecessary side effects; (3) identifying additional inhibitors downstream of the Wnt/β-catenin signaling pathway; and (4) considering CGA structural modifications to improve the pharmacological profile and/or the affinity for β-catenin and LRP6. (5) In order to elucidate the relationship between ROS production and cell death in CGA-treated cells, other experiments should be performed. Treatments with antioxidants that block or mitigate ROS production would allow us to determine whether ROS is a mediator of this biological effect. 

## Figures and Tables

**Table 1:** Affinity and interactions of CGA and validation compounds (iCRT14 and C2) with β-catenin at the biding (US) and allosteric (AS) sites.

Protein	Compound	Vina Score (Kcal/mol)	Protein-Ligand Interactions		
			Hydrogen Bonds	π Interactions	Hydrophobic Interactions
β-catenin AS	CGA	−5.5	ARG587		
			ASP583		
			HIS585		
			PRO521		
			HIS524		
			ALA525		
			GLN623		
	C2	−4.6	ARG587		
			ASP583		
			VAL584		
β-catenin US	CGA	−6.5	ASN430	HIS470	ARG469
			LYS435		
			ARG474		
			ASN516		
			SER473		
	iCRT14	−5.3	ARG469	ARG474 ARG515	
			HIS470		
			LYS508		

**Table 2:** Affinity and interactions of CGA and Niclosamide with LRP6.

Protein	Compound	Vina Score (Kcal/mol)	Protein–Ligand Interactions		
			Hydrogen Bonds	π Interactions	Hydrophobic Interactions
LRP6	CGA	−6.3	SER665 GLU708		GLU708
					ARG751
					TRP767
					LEU810
					PHE836
	Niclosamide	−6.3	ASP811 HIS834	HIS834	TRP767
					LEU810
					PHE836

**Table 3:** Docking parameters.

Protein Subsite	Center of the Grid	Size	Exhaustiveness
β-catenin US	x = 11.527, y = 22.308, z = 62.347	17 Å^3^	20
β-catenin AS	x = 2.805, y = 14.864, z = 79.543	17 Å^3^	20
LRP6 E3	x = 26.038, y = 5.167, z = −15.270	24 Å^3^	20

## Data Availability

Data is contained within the article.
